# Femtosecond laser-assisted fabrication of piezoelectrically actuated crystalline quartz-based MEMS resonators

**DOI:** 10.1038/s41378-023-00511-5

**Published:** 2023-03-30

**Authors:** John Linden, Neta Melech, Igor Sakaev, Ofer Fogel, Slava Krylov, David Nuttman, Zeev Zalevsky, Marina Sirota

**Affiliations:** 1 Department of Engineering, Bar Ilan University, Ramat Gan, Israel; 2 Research and Incubation, ERC, KLA, Yavne, Israel; 3 School of Mechanical Engineering, Faculty of Engineering, Tel-Aviv University, Tel Aviv, Israel; 4 Engineering Department, TAMAM, IAI, Yehud, Israel

**Keywords:** NEMS, Structural properties, Electrical and electronic engineering

## Abstract

A novel technology for the precise fabrication of quartz resonators for MEMS applications is introduced. This approach is based on the laser-induced chemical etching of quartz. The main processing steps include femtosecond UV laser treatment of a Cr-Au-coated Z-cut alpha quartz wafer, followed by wet etching. The laser-patterned Cr-Au coating serves as an etch mask and is used to form electrodes for piezoelectric actuation. This fabrication approach does not alter the quartz’s crystalline structure or its piezo-electric properties. The formation of defects, which is common in laser micromachined quartz, is prevented by optimized process parameters and by controlling the temporal behavior of the laser-matter interactions. The process does not involve any lithography and allows for high geometric design flexibility. Several configurations of piezoelectrically actuated beam-type resonators were fabricated using relatively mild wet etching conditions, and their functionality was experimentally demonstrated. The devices are distinguished from prior efforts by the reduced surface roughness and improved wall profiles of the fabricated quartz structures.

## Introduction

Crystalline quartz is an exceptional material distinguished by its unique ability to maintain stable material properties within a wide temperature range. Resonant quartz devices realize high mechanical quality factors and offer frequency-temperature stability of approximately 0.6 ppm per degree centigrade^1,2^. Although significant progress has been achieved recently toward improving the frequency-temperature stability of Si resonators^3^, quartz remains a very popular and attractive material due to its unique piezoelectric properties. This feature allows for the integration of both actuating and sensing electrodes within the same resonating element rather than as separate entities, as is required for electrostatically actuated Si devices. This benefit may reduce alignment-related errors in high-end sensors. These features have favored the utilization of quartz as a substrate for the fabrication of different types of meso and microscale (MEMS) devices, such as tuning fork resonators for timing applications and frequency control and as mass, gas, and humidity sensors^4–7^. Quartz is also indispensable in high-end inertial sensors such as resonant accelerometers and gyroscopes, pressure sensors, electric and magnetic field detectors, and ultraprecise chemical scales (quartz crystal microbalance)^8–10^. In all these devices, the performance quality is measured by analyzing the variations in the structure’s eigenfrequencies under the stress of external stimuli^11,12^. Manufacturing technologies compatible with larger-scale devices, such as quartz clocks or quartz microbalances for microscale mass sensing, are based on traditional machining methods and are well established. However, these techniques cannot be directly implemented for the fabrication of microscale devices. Thus, the development of new, more suitable approaches is needed.

Several existing fabrication techniques for MEMS devices made of quartz are summarized in Table 1. Traditional machining methods (such as computer numerical control, CNC), while well established, are not suitable for the fabrication of microdevices, mainly due to their inability to achieve the desired structural dimensions and quality. Poor attributes in the resulting structures, such as high surface roughness and microcracks, lead to the deterioration of device performance and prevent the process implementation of large-scale high-end systems. While various finishing methods are routinely available on the macro scale, such as for hemispherical quartz gyros^13^, their implementation in microdevices may negatively affect manufacturability and cost (and in many cases is not even possible).

**Table 1:** Existing methods used for the fabrication of quartz MEMS devices

Fabrication method	Advantages	Disadvantages	Limitations
*Traditional CNC machining*	No reliance on crystal orientation	High surface roughness, microcracks	Not suitable for microscale
*Wet isotropic/anisotropic etching*^52,53^	Simplicity, reliability; consistent process control; Suitable for mass production	Multistage process; Reliance on crystal orientation; Etching rate nonuniformity across the wafer Nonuniformity of the cross-sectional profile; Requires lithography and masking	Complex prototyping requiring mask development
*Deep Reactive Ion Etching (DRIE)*^14^	No reliance on crystal orientation; Low surface roughness; Suitable for mass production	A low width-to-depth aspect ratio of etched structures; Requires lithography and masking	Suitable for low aspect ratio structures or Quartz-on-substrate devices
*Laser ablation micromachining*^19,54,55^	No reliance on crystal orientation; Mask-less process; High aspect ratio structures	Cracks, surface damage Stress; Alters surface material structure via transformation	Nonlinear effects can be disruptive; Surface defects affecting the device’s natural frequency, deteriorating device performance
*Femtosecond Laser-induced chemical etching* *(FLICE)*^22,56,57^	No reliance on crystal orientation; Mask-less process; High aspect ratio structures	May create amorphous regions near the processed surface	Has never been used for functional Quartz actuators

Wet etching presents an efficient process for the fabrication of quartz structures, especially for double-sided etching of samples with thicknesses spanning tens or hundreds of microns. Quartz wet etching is generally performed in saturated ammonium bifluoride (NH_4_HF_2_) or in BOE (Buffered Oxide Etchant), which is a mixture of NH_4_F:HF at temperatures of 80 °C or higher. The etchants attack the $\hat z$-plane as well as several other quartz orientations, while minimally affecting other planes. Nevertheless, strong anisotropy of the etching due to the trigonal symmetry of quartz crystal inevitably results in structures with complex nonsymmetric geometries and oblique nonorthogonal facets, which makes the generation of desired shapes difficult.

Deep reactive ion etching (DRIE) is a promising and intensively researched technique^14–16^ that allows for the wafer-level fabrication of microscale structures with relatively smooth vertical sidewalls. Several fabrication facilities offer DRIE quartz etching services. However, the overall depth and aspect ratio of the resulting structures fabricated by this process is limited, it is relatively slow, and it is less suitable for the fabrication of devices with a thickness in the tens of micrometers or hundreds of micrometers range. In addition, similar to common MEMS processes, quartz DRIE requires lithography and masking, making it less suitable for prototyping efforts.

Femtosecond (fs) laser-induced chemical etching (FLICE) is a promising emerging method for the micromachining of transparent solid materials. The FLICE method for preparing three-dimensional (3D) structures comprises two steps: first, photomodification of the transparent material by irradiating a train of pulses with a focused femtosecond laser beam; second, immersion of the irradiated material in etching solutions for a specified duration. For example, the fabrication of 3D microchannels with the FLICE method has been realized for silica^17–19^, sapphire^20^, and borosilicate glasses^21^. In addition, laser-induced chemical etching has been applied for quartz crystal surface structuring^22^ or in-quartz microchannel fabrication^21^. However, the method has not been used for quartz “through-cut” or actuated functional device manufacturing. Moreover, the reported laser-assisted etching technologies^23,24^ did not allow quartz to retain its excellent piezoelectric properties due to amorphous regions created by laser-matter interactions.

Here, we report an application of a refined FLICE process with the general goal of establishing a method that allows for the micromachining of quartz structures with desired shapes and dimensions while preserving the crystalline structure and minimizing postproduction defects. We found that optimizing the parameters of an ultrashort laser (~270 femtosecond pulse width) at 343 nm results in quartz bulk modification without changing the quartz crystalline structure while still producing the desired etching selectivity. The suggested fabrication framework preserves all the advantages of laser micromachining, such as fast (lithography-free) prototyping, the ability to create structures of desired symmetric geometries through wafer-scale processing, and the patterning of metal coating by the laser, without negatively impacting the quartz crystalline structure. Except for the metal layer deposition and wet etching, this fabrication process is executed using one laser tool and without clean-room requirements. This approach was found to be very efficient and convenient for rapid prototyping during device development, where various configurations can be fabricated, tested, and optimized simultaneously in a short time. The FLICE method may also be comparable to the overall time for lithography-based processes, where ~15 min is required to process an area of 150 mm^2^.

These features collectively demonstrate that the suggested FLICE process is especially suitable for the fabrication of functional, piezoelectrically actuated resonant MEMS devices for high-end sensing applications.

## Materials and methods

### Quartz structure manufacturing process (FLICE)

The starting material was Z-cut double-side polished quartz wafers^25^. Z-cut quartz is widely used for the fabrication of microactuators, microsensors, and other force-sensitive devices that exploit flexural modes of vibrations. In addition, *Z*-cut quartz conveniently has a high wet etch rate through the *Z* plane. The wafers were 4″ in diameter, with thicknesses of 100 µm and 300 µm, and with a primary flat oriented in the *X*-cut direction for alignment purposes.

To create a functional resonator shaped from quartz, the following conditions must be achieved in perfect cohesion:

- Full device geometry
- Precise quartz crystal orientation with respect to the geometry
- Electrode positioning concerning the quartz crystallographic direction

**Fig. 1:**
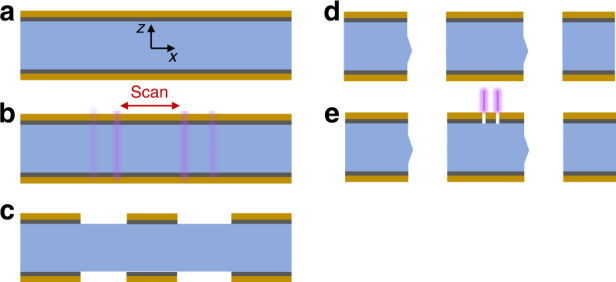
Proposed FLICE process flow for a substrate of 100 μm quartz wafer. **a** quartz wafer is coated on both sides with the Cr/Au metallic layer, **b** laser ablation of the metallic layer and the quartz hard-mask formation, **c** wafer state after laser ablation, **d** wet etching of quartz, and **e** electrode formation by laser ablation

With these requirements in mind, the FLICE-based fabrication method suitable for quartz device manufacturing and detailed in Fig. 1 was implemented.

The process starts with a PVD coating of the wafer on both sides with a Cr/Au:20 nm/200 nm thick layer. At this first stage of fabrication, the Cr-Au coating serves as the mask for the wet etching process. Next, the Cr/Au film is ablated with the laser, defining the electrode patterns. Then, additional laser scans are repeated with lower pulse energy to enhance wet etch rate selectivity. After, the wafer undergoes a wet etch process. The laser-treated area is etched until the desired thickness is reached by timing the immersion in the etchant. After the first etch process is completed, the metal coating is chemically removed and the entire wafer is recoated with a new Cr/Au layer. This coating serves as both the etch mask and the base conductive layer for the actuating and sensing electrodes. In the next stage, laser ablation of the Cr/Au layer is followed by low-energy laser scans for selectivity of future etching of the trenches. Then, the second wet etch process is performed, resulting in a through-cut of the wafer and finalizing the geometry of the stand-alone quartz structure. In the final stage, the actuation electrodes and the conductors at the wafer top surface are applied by patterning with a laser ablation Cr/Au layer with laser ablation.

**Fig. 2:**
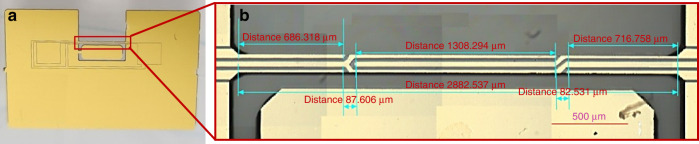
Full functional device overview. **a** View of the whole quartz device with electrodes and contact pads. **b** Enlarged image of electrodes on the resonating quartz beam (2890 × 120 × 100 µm)

The final beam-type device with the electrodes is shown in Fig. 2.

### Laser scanning

The common laser parameters for FLICE processes are ultrashort pulses with pulse durations varying between several hundred femtoseconds and up to several picoseconds. In this process, the laser wavelength is usually in the near IR regime at either 1030 nm or 1064 nm.

The ultrashort pulses reach extremely high intensities in the substrate, approximately $1e^{12}\,({\rm{W/cm}}^2)$. At this power density, nonlinear multiphoton absorption occurs, leading to material alterations from the nucleation of defect atomic states to the rupturing of molecular bonds. A thorough analysis of the various phenomena involved can be found in the literature^20,26–28^. By using the third harmonic (THG) of the initial wavelength, we further increase the incident photon energy, which proved to be a critical factor in the process developed by this study. As a result of these physical and chemical modifications associated with multiphoton absorption, the irradiated areas acquire an increased solubility to acids.

**Fig. 3:**
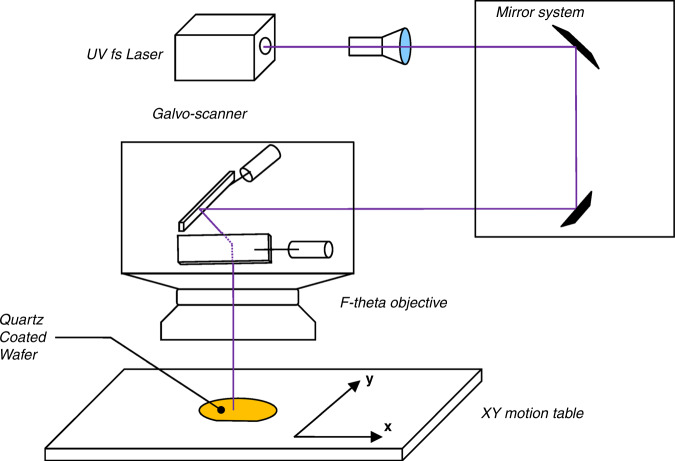
Experimental Setup. Schematics of laser propagation path to substrate allowing scanning accuracies of several microns. The XY motion table also included a rotation axis for the alignment of the wafer flat primary

**Fig. 4:**
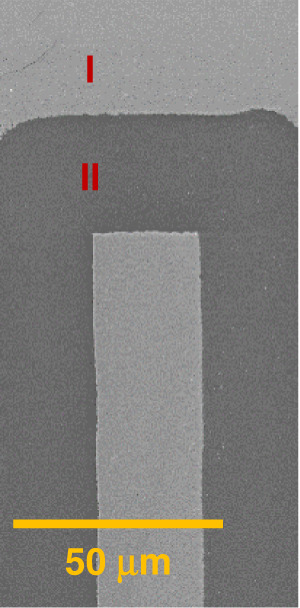
Selective Ablation Mask. SEM images of Cr/Au coating ablation patterns. The Au-coated bright area (I) was not scanned; in the dark area (II), Au was removed by ablation

The laser used was a Tangerine model by Amplitude-Laser^29^ combined with a THG option capable of producing pulsewidths of 270 femtoseconds up to 10 picoseconds. This creates output pulses of 343 nm wavelength with pulse energies up to 40 μJ at a pulse repetition rate of 175 kHz. The apparatus used for scanning the samples from the laser output consisted of several low group delay dispersion mirrors (GGD) and a Newson galvanometer scanner with an f-theta lens of 100 mm (see Fig. 3). This setup produces a Gaussian beam spot size of ≈20 μm. At the maximum pulse energy, peak powers of ≈$10e^{13}\,\left( {\frac{{\rm{W}}}{{{\rm{cm}}^2}}} \right)$ can be reached on the substrate, resulting in undesired direct cold ablation of the substrate material^30^. For pulse energies of ~3 $\upmu {{{{{\rm{J}}}}}},\,7e^{12}\left( {\frac{{\rm{W}}}{{{\rm{cm}}^2}}} \right)$ the Au/Cr coating is ablated without damaging the underlying substrate (see Fig. 4). Following the ablation of the coating layer, up to 50 additional scans over the exposed wafer surface with a pulse energy of 1 μJ and a pulse-to-pulse pitch of 3 μm (at a pulse repetition rate of 44 kHz) generate the desired defects, resulting in etch selectivity. It is worth noting that the laser spot size determines the critical dimension of the device. If smaller critical dimensions are required for a device, the laser fluence/peak power should be maintained so as not to contribute to undesired nonlinear propagation phenomena within the substrate.

### Laser-matter interactions with quartz

We further explain the material modifications according to possible interactions that occur when a femtosecond laser beam interacts with a quartz crystal. This interaction consists of a variety of physical processes depending on the laser parameters and the crystal properties. As stated, one of the most important interactions is nonlinear absorption. The efficiency of this process is determined by the material’s nonlinear susceptibility, which is a measure of its ability to interact with the laser field. Nonlinear absorption can also lead to the formation of defects and color centers in the crystal, which affect its optical and electrical properties^31,32^.

Considering the laser parameters as mentioned above, with the third-order nonlinear susceptibility of the quartz crystal as $\chi ^{\left( 3 \right)}\sim 4.2 \times 10^{ - 22}$, we define:

1 $$\begin{array}{*{20}{c}} {n_2 = \frac{{3\chi ^{\left( 3 \right)}}}{{4{\it{\epsilon }}_0cn^2}}} \end{array}$$

where *n*_2_ is the nonlinear refractive index of the crystal, $\lambda _o$ is the laser wavelength, *c* is the speed of light in a vacuum, ${\it{\epsilon }}_0$ is the vacuum permittivity, and *n* is the refractive index of the crystal.

Since our peak intensity is ~47,005 ${\rm{MW/mm}}^2$, we exceed the critical power for self-focusing given by:

2 $$\begin{array}{*{20}{c}} {P_{cr} = \frac{{\pi \left( {0.61} \right)^2\lambda _o^2}}{{8n_on_2}}} \end{array}$$

This yields a factor of ~17. We note that most likely only above a factor of ~100 will the beam break-up occur, while the self-focusing distance will be reached at a depth of:

3 $$\begin{array}{*{20}{c}} {z_{sf} = \frac{{\omega _0}}{{\sqrt {\frac{{2n_2I}}{n}} }} \approx 130\,\upmu {\rm{m}}} \end{array}$$

**Fig. 5:**
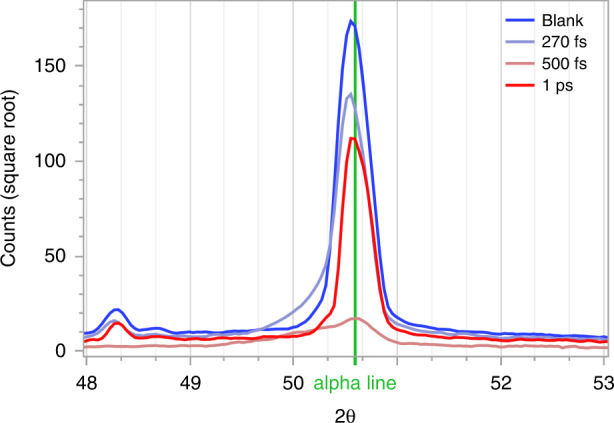
Crystallography Measurement Results. XRD lines of quartz samples treated by UV laser with different pulse durations compared to a blank wafer with no processing. Settings: laser pulse repetition rate (PRR) of 43 kHz, 100 mm/s scanning velocity, 8 µJ pulse energy

In our case, this thickness is beyond the thickness of the quartz wafer, so we do not reach the full limit of the self-focusing; however, this process still results in a converging beam that reaches intensities exceeding 20 ${\rm{TW/cm}}^2$ at a depth of ~88 μm within the quartz wafer. At these intensities, a small region within the focal volume is very rapidly ionized by the combined action of avalanche and multiphoton processes in only a few optical cycles, causing dielectric breakdown and creating an ionized solid-state density plasma^33^. This plasma effectively absorbs ~60% of the incident energy. Considering a spherical plasma volume defined by the spot size at 88 µm depth with a skin depth of ~60 nm, we calculate the absorbed energy density as translating to a pressure of ~400 GPa ^34,35^. This produces a significant shockwave within the substrate that is significantly above Young’s modulus of the quartz wafer (97.2 GPa), thus resulting in localized nanometer voids in the surrounding radius where the shockwave is comparable to the absorbed energy: $\left( {\frac{4}{3}} \right)\pi R^3p_0 = E_{abs}$, where $p_0$ is the Young’s modulus. In our case, this volumetric radius approaches ~1.2 µm, which supports the XRD results in Fig. 5 showing that even if nanometer-scaled voids are formed, this does not result in an amorphous structure but does induce stresses on the crystal lattice structure.

An additional process that can cause distortions in the crystal stoichiometry is piezoelectric excitation, whereby the laser pulse mechanically deforms the crystal and thus induces an electric charge^36–39^. This process is governed by the piezoelectric coefficient of the crystal, which determines the magnitude of the electric field generated by the deformation. Since significant absorptance occurs only at the plasma formation site, this effect is dwarfed by the induced shockwave; however, it is nonetheless considered due to be a known effect.

From this calculation, we derive that the stress induced by the shockwave is the most significant factor that results in the desired material defects as further described below.

**Fig. 6:**
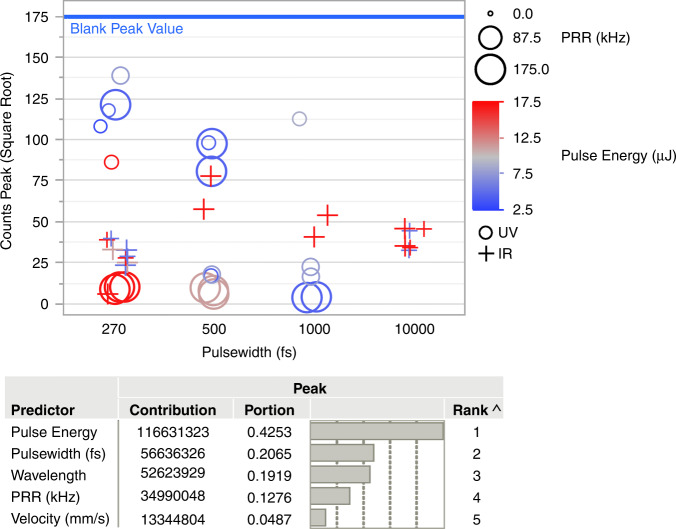
FLICE Design of Experiement Results. Alpha peak values of 2θ = 50.67 for various laser powers (color scale), pulse widths (y-axis), wavelengths (symbol), and PRR (size) as measured with XRD. The upper deep blue line shows the value for quartz that was nontreated with a laser (valued at 175). The table below displays the list of predictors with their respective contributions and ranks to a bootstrap forest partitioning model

Quartz crystal functional devices essentially rely on quartz’s piezoelectric properties. To characterize the post-processed quartz structure and specifically to ensure that the process did not damage the crystalline structure of the material, we performed X-ray diffraction (XRD) tests. The results of XRD measurements are shown in Figs. 5 and 6.

XRD spectra of quartz samples treated by laser demonstrate a pronounced peak at 2θ = 50.67, indicative of the quartz (2)003 plane. The XRD patterns indicate the presence of only crystalline material. However, some peak broadening and slight shifts can be seen in the XRD pattern, especially after 500 fs and 1 ps pulsewidths, as shown in Fig. 5. Notably, the peak at 1 ps surpasses that at 500 fs; however, there is also a noticeable change in the crystalline plane with a peak appearing at 2θ = 38 (not illustrated). A broader comparison in Fig. 6 shows the advantage of using a lower powered UV wavelength at a short pulsewidth of 270 fs to maintain values that better resemble the unprocessed crystalline material. Further apparent in Fig. 6 is the increased damage to the crystalline structure generated by the IR wavelength, as noted by a low peak count value even at 270 fs pulsewidth. This effect contributes to the preference for UV ultrashort pulses at low repetition rates for FLICE fabrication of quartz MEMS. We attribute these effects of pulsewidth and pulse energy directly to the ionization threshold, as has been described thoroughly^34^.

We attribute the peak broadening and/or shifts seen in XRD analysis to the strain^40^ induced by the laser-matter interactions, as described above. This in turn may result in several modifications to the substrate:

1. Changing the crystallite/diffracting domain size.
2. Crystal lattice distortion (microstrains) induces dislocations and creates concentration gradients of atomic structures, resulting in distortions in stoichiometry.
3. Formation of structural defects that do not align with the full crystalline lattice.

Figure 6 indicates that both homogeneous and inhomogeneous strains in the samples result in XRD peak broadening and shifting. These effects are more pronounced at pulsewidths longer than 500 fs and for higher laser powers. Nevertheless, we conclude that the sample has preserved its crystalline structure after the process of applying UV energy at ultrashort femtosecond pulsed scanning. This observation is consistent with the results of the actuation testing of the devices. These precise localized and controlled laser-induced strains in the quartz are the primary cause of etching selectivity.

### Chemical etching of quartz

The next significant step in the process is the etching of the substrate. We used a mixture of hydrofluoric acid (HF) and ammonium fluoride (NH_4_F) to etch the quartz after laser treatment. The ratio NH_4_F:HF and solution temperature were changed to create a membrane of the required thickness or through-cut trenches. We found that the optimal NH_4_F:HF ratio lies in the range between 4:1 and 1:1; the etching temperature was held between 40 and 60 °C. The selected parameters allowed controlled etching with an etching rate of 20–40 µm/h, depending on the temperature and NH_4_F:HF ratio. We observed that slower etching resulted in better surface quality, but this trend requires further investigation.

**Fig. 7:**
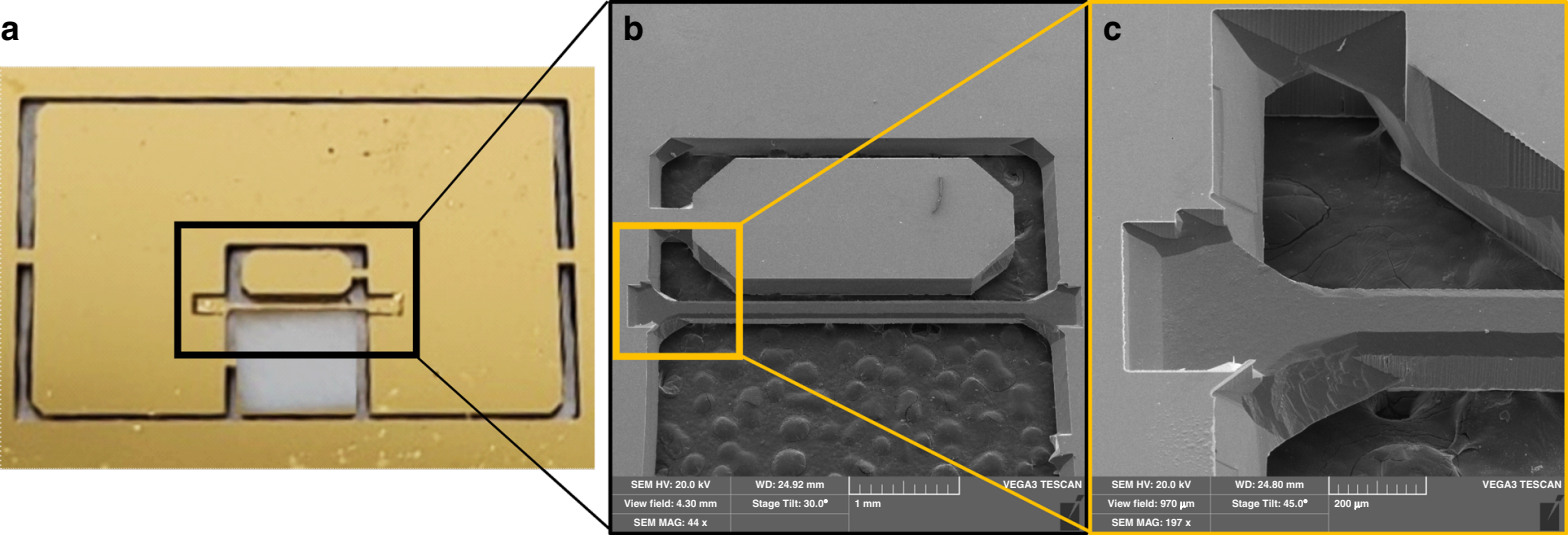
Quartz resonators are fabricated by the FLICE process. **a** Overview image of the entire device. The beam is oriented in the y direction. **b** Zoomed-in tilted (30°) scanning electron microscopy (SEM) image of an 80 µm wide and 100 µm thick beam fabricated from a 300 µm thick wafer. The beam position is in the middle of the wafer, and the etch depth is 100 µm. **c** Zoomed-in tilted (45°) image of the beam near the attachment point. The etched lateral beam surface (sidewall) is visible

**Fig. 8:**
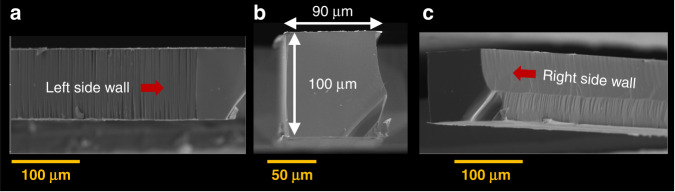
SEM images of the quartz beam fabricated by the FLICE process from a 100 µm thick wafer. **a** Side view from the left of the beam. **b** Cross-sectional view of the 90 µm wide 100 µm thick beam. **c** Side view from the right of the beam with “facet” corresponding to the crystallographic structure

While there are many types of quartz resonant devices, most quartz sensors, such as accelerometers and pressure sensors, use double-ended tuning forks or double-clamped single beams^41,42^; as such, we have applied those designs in this fabrication experiment. Examples of the quartz shapes as fabricated by our FLICE process are presented in Figs. 7 and 8.

Quality inspection of the final shape was carried out with a Zeiss Differential Interference Contrast (DIC-Nomarski) microscope with a magnification of 200× and with an atomic force microscope. The measured roughness of the upper surface etched area (Fig. 7) was less than 200 nm.

The influence of the laser treatment on the etch process is revealed by two phenomena, namely enhancement of the etching and improvement of the etched surface quality.

It seems that the laser treatment did not significantly influence the etch rate. A direct comparison of etch rates with those reported in other studies^6^ would be invalid since very different etching parameters were presented in those works. However, we argue that laser irradiation noticeably improves the process by making the propagation of the etched plane homogeneous and parallel to the wafer surface without creating angled membranes in the crystallographic plane, as are created in other wet etch processes^20^.

**Fig. 9:**
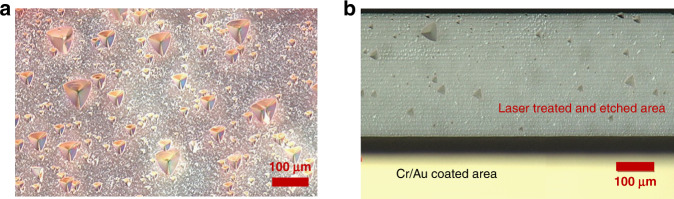
DIC microscopic images of wet etched quartz surfaces, etch depth was 100 µm. **a** Quartz surface after 4 h of etching without laser treatment before etching. **b** Quartz surface after 4 h of wet etching under the same conditions as in (**a**) and after laser treatment before etching

A comparison of etched quartz surfaces that were and were not irradiated is shown in Fig. 9. Therefore, this FLICE fabrication method allows us to achieve better surface quality with the same wet etch parameters.

Following the process in Fig. 1, the second coating of the wafer with Cr/Au was applied for electrode fabrication by laser direct writing on the Cr/Au layer in the defined regions. The final beam-type device with the electrodes is shown in Fig. 2.

### Role of process parameters

The primary features of the fabrication method are ensuring the device’s integrity and minimizing mechanical defects due to the stress and strain created during the fabrication process.

Laser micromachining can cause defects that appear throughout the quartz wafer (top, middle, or bottom) due to undesired stress formation from self-focusing and other phenomena, as described in ref. ^43^, resulting in subsequent cracking of the processed material^44–47^.

By focusing the process parameters to only ablate the top layer and performing consecutive scanning to instigate structural defects without the presence of material ablation, no plasma is formed, and thus no significant stress is created in the substrate. This approach mitigates the defects to only those desired, resulting in selectivity for the wet-etch process. It is important to note that the proposed method shows good robustness to small process variations such as pulse-to-pulse stability and additional scanning velocity/repetitions.

### Functional testing

Following the FLICE process to fabricate single-crystal quartz MEMS resonators comprising several configurations, the devices were operated in air and their functionality was demonstrated. The dynamic responses, vibrational amplitudes, and Q-factors were measured. The experimental resonant frequencies and amplitudes were compared to values predicted by a finite element (FE) model.

**Fig. 10:**
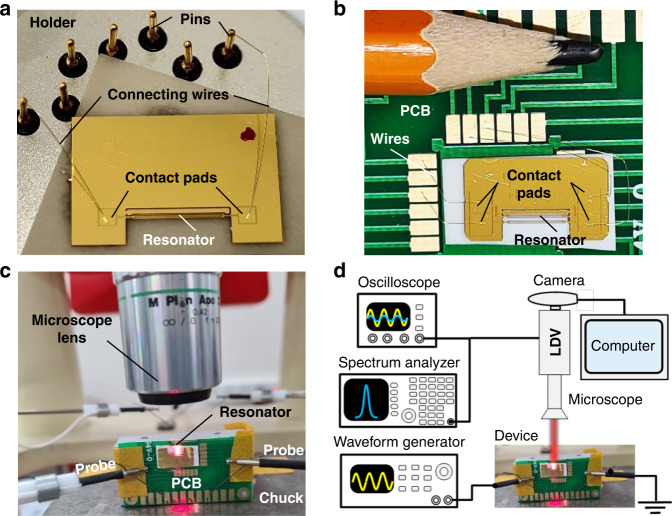
Experimental setup for resonance measurements. **a** Wire-bonded quartz resonator chip mounted on a metallic holder. Connecting wires are attached to the holder’s pins. **b** The chip attached to the PCB with relative scale to pencil. **c** The PCB with the quartz chip is vertically mounted on the wafer prober chuck. The device is electrically connected by microprobes. **d** Schematics of the electrical connections

To connect the actuating electrodes to the voltage sources, two mounting approaches were implemented. In the first arrangement, chips with crystal resonators were attached to a metallic holder with conductive pins. One side of each of the contact wires was wire-bonded to the contact pads on the chip, while the other side was connected to the pins, as in Fig. 10a. These pins were connected to the voltage source using mechanical clips. In the second arrangement, the connecting wires were bonded to the chip pads and the custom-built PCB pads, as shown in Fig. 10b. These PCB pads were connected to the voltage source by micromanipulators (probes). The metallic holders or PCBs were placed on the wafer prober chuck in such a way that the plane of the chip was perpendicular to the chuck plane and the sidewall of the beam was visible through the microscope, as shown in Fig. 10c.

The functionality tests included measurements of the resonant frequencies associated with the fundamental in-plane (parallel to the chip plane) mode of vibration, amplitudes at the resonant frequencies, and quality factors. All experiments were conducted under ambient air conditions. Before operation, the resonator dimensions were measured with an optical microscope. The fabricated device’s geometry dimensions were within 3% of the designed specifications.

In all tests, one of the resonator’s electrodes was grounded. An AC voltage supplied by a two-channel arbitrary function generator (Tektronix AFG3022C) was applied to the second electrode. To induce the resonant responses of the beams, a fast periodic chirp was implemented to first locate the frequencies, followed by a slower linear chirp lasting 30 s, where the voltage signal frequency, *f*, was driven from 70 kHz to 78 kHz in the vicinity of the calculated baseline fundamental mode frequency of *f*_0_ ≈ 76 kHz. The actuating voltage was modulated between 1 V and 5 V. The input signal was monitored in real-time by a four-channel oscilloscope (Keysight InfiniiVision DSOX2004A). The dynamic responses of the resonators were registered using a single-point laser Doppler vibrometer (LDV, by Polytec GmbH) operated in velocity mode and equipped with an OFV-551-1 sensor head, an OFV-5000 controller, and a VD-06 velocity decoder. The LDV detector beam collimated by a microscope (Navitar with the long working distance Mitutoyo ×20 lens) was focused on the middle of the resonator beam. We note that the vertical positioning of the chip at the edge of the PCB conveniently allows direct measurement of the beam’s velocity response by LDV^48^. Signals from the LDV were input into an oscilloscope and a real-time spectrum analyzer (Tektronix RSA3000B). The acquired spectral data were fitted numerically using a Lorentzian function, and the corresponding resonant frequencies and quality factors were obtained. Since the LDV was operated in velocity mode, the amplitudes *u*_0_ of the nearly time-harmonic responses were recalculated using the known expression, where *V*_LDV_ is the vibrometer analog output (in V), *α* = 10 mm/s is the LDV conversion factor and *f* is the output signal frequency.

## Results and discussion

Quartz resonators were fabricated with nominal dimensions of *L* = 2650 ± 75 µm and *x* = 90 ± 3 µm, and height of *h* = 100 ± 3 µm. The resonant frequency vibrational mode was measured in the *xy* plane of the quartz crystal, parallel to the wafer surface. This was compared to an FE model of the resonator as designed in COMSOL. The beam was modeled using 24,872 three-dimensional solid brick-type and tetrahedron elements. For the material properties, the full matrices of the quartz stiffness and piezoelectric coefficients were used^49,50^, and the density of quartz was set as *ρ* = 2650 kg/m^3^.

**Fig. 11:**
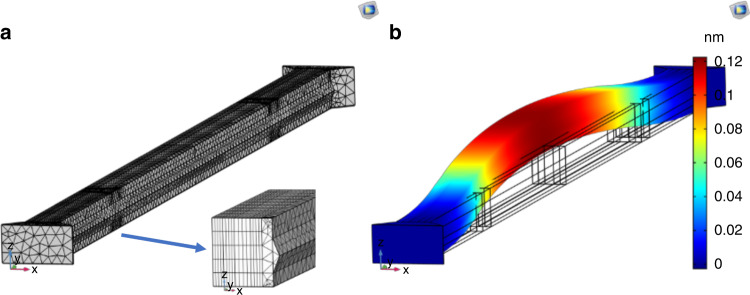
Simulation of Quartz Resonator Beams. **a** Close-up of the mesh used for modal analysis and static deflection measurements with a zoom of cross-section, as simulated with COMSOL software. The model contained the real geometry of the tie, including oblique facets. **b** Static deflection in the in-plane direction (x-axis)

Linear modal analysis was used to calculate the resonator beam’s fundamental frequency mode, as shown in Fig. 11a, and was found to be *f*_0_ = 73436 Hz. Coupled static electromechanical analysis was used to estimate the static deflections of the piezoelectrically actuated beam. A static DC voltage of *V*_dc_ = 5 V was used. The FE model result displacement is shown in Fig. 11b.

**Fig. 12:**
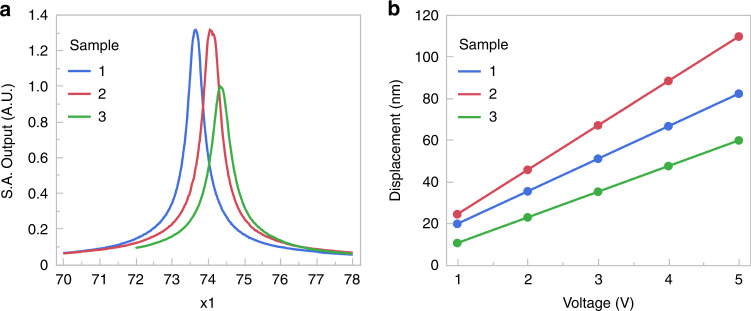
Measured Responses from LDV. **a** Spectral response of the beams vibrating in the fundamental in-plane mode. Different curves correspond to the different sample resonators of the same nominal dimensions. **b** Resonant midpoint amplitudes, in terms of the beam’s midpoint displacements, vs. actuating voltage dependence of several sample resonators

The spectral response of the three samples’ resonator beams at the actuating voltage of *V*_ac_ = 5 V is shown in Fig. 12a. A linear Lorentzian response was observed in all cases. The linearity of the resonator response is illustrated in Fig. 12b, where the voltage-amplitude dependence is shown. The results are summarized in Table 2. The average resonance frequency of all the resonators is 74.02 kHz with a standard deviation of 0.35 kHz, which differs from the calculated value by 0.47%. The variation between the different resonators is mainly attributed to the beam’s dimensions, geometric measurement errors, and undetected defects in the resonators.

**Table 2:** Experimental results summary

Sample number	1	2	3
Resonant frequency (kHz)	73.65	74.06	74.36
Q factor	208	233	169
Resonant peak displacement (nm), measured (calculated)	~82 (95)	~109 (104)	~59 (62)

The calculations of the natural frequencies and modes of the devices were performed under the consideration of a steady, time-independent force. At the resonance, the amplitude is equal to the product of the static deflection and the *Q*-factor, such that the amplitudes were estimated by dividing the measured amplitudes by the measured *Q*-factor and comparing the resulting values with those given by a static displacement method^51^. This comparison is seen in the last row of Table 2. While the resulting q-factors are relatively low, the accuracy of the simulated model with respect to the measurement data demonstrates the quality and integrity of the fabrication methods presented in this study. The manufacturing approach further presents a method that can easily be implemented from prototyping to production of high-quality quartz MEMS devices with high confidence. Although the systematic investigation of the q-factor is beyond the scope of this work, it will be the focus of future efforts toward optimizing the device’s geometry for superior performance.

## Conclusions

In this study, we demonstrated a method for fabricating piezoelectrically actuated crystalline quartz-based MEMS resonators by femtosecond laser-induced chemical etching. We proved our method’s feasibility and integrity for prototyping functional MEMS devices. We reviewed the mechanisms underlying the viability of the method by analyzing the crystalline structural modifications from laser-matter interactions. We determined that with ultrashort pulses (on the order of hundreds of femtoseconds at 343 nm wavelengths), fine structural qualities maintain their original monocrystalline orientation, resulting in a high experimental resonance that closely matched simulated results under ideal calculations. There exist numerous types of sensors, such as resonant accelerometers, magnetic and electric field sensors, pressure sensors, and others, that can be based on the double-ended tuning fork (DETF) or quartz tuning fork (QTF). Therefore, in this study, we fabricated and tested devices comprising several of these configurations and propose that our suggested approach can be implemented for the fabrication of all the aforementioned devices.

The combination of methods used for fabricating the quartz-based MEMS device presents an advancement that paves the way for implementing single-crystal quartz into MEMS components. With the paramount advantages that quartz offers relative to other common MEMS materials, functional components with greater stability, higher accuracy, and increased sensitivity are possible. The capability for full integration of a MEMS device with high-quality machining and an embedded electronic circuit positions quartz as an increasingly promising material for future MEMS development.
